# Epitope tag-specific differences in the detection of COSA-1 marked crossover sites in *C. elegans* spermatocytes

**DOI:** 10.17912/micropub.biology.000724

**Published:** 2023-01-06

**Authors:** Cori K. Cahoon, Celja J. Uebel, Anne M. Villeneuve, Diana E. Libuda

**Affiliations:** 1 Institute of Molecular Biology, Department of Biology, University of Oregon, Eugene, OR, USA; 2 Stanford University School of Medicine, Departments of Developmental Biology and Genetics, Stanford, CA, USA

## Abstract

Nascent crossover sites in *C. elegans* meiocytes can be cytologically detected using epitope-tagged versions of the pro-crossover protein COSA-1. In spermatocytes, differences exist between cytologically-detected and genetically-detected double crossover rates. Here, we examine nascent crossovers using both GFP- and OLLAS-tagged COSA-1. Similar to previous work, we find that most late pachytene spermatocytes display 5 COSA-1 foci, indicating one crossover per autosome bivalent. However, we detected more nuclei with >5 COSA-1 foci using OLLAS::COSA-1, reflecting some bivalents having 2 COSA-1 foci. These results demonstrate tag-specific differences in the detection of COSA-1 marked nascent crossovers in spermatocytes.

**Figure 1. COSA-1 foci in late pachytene spermatocytes.**
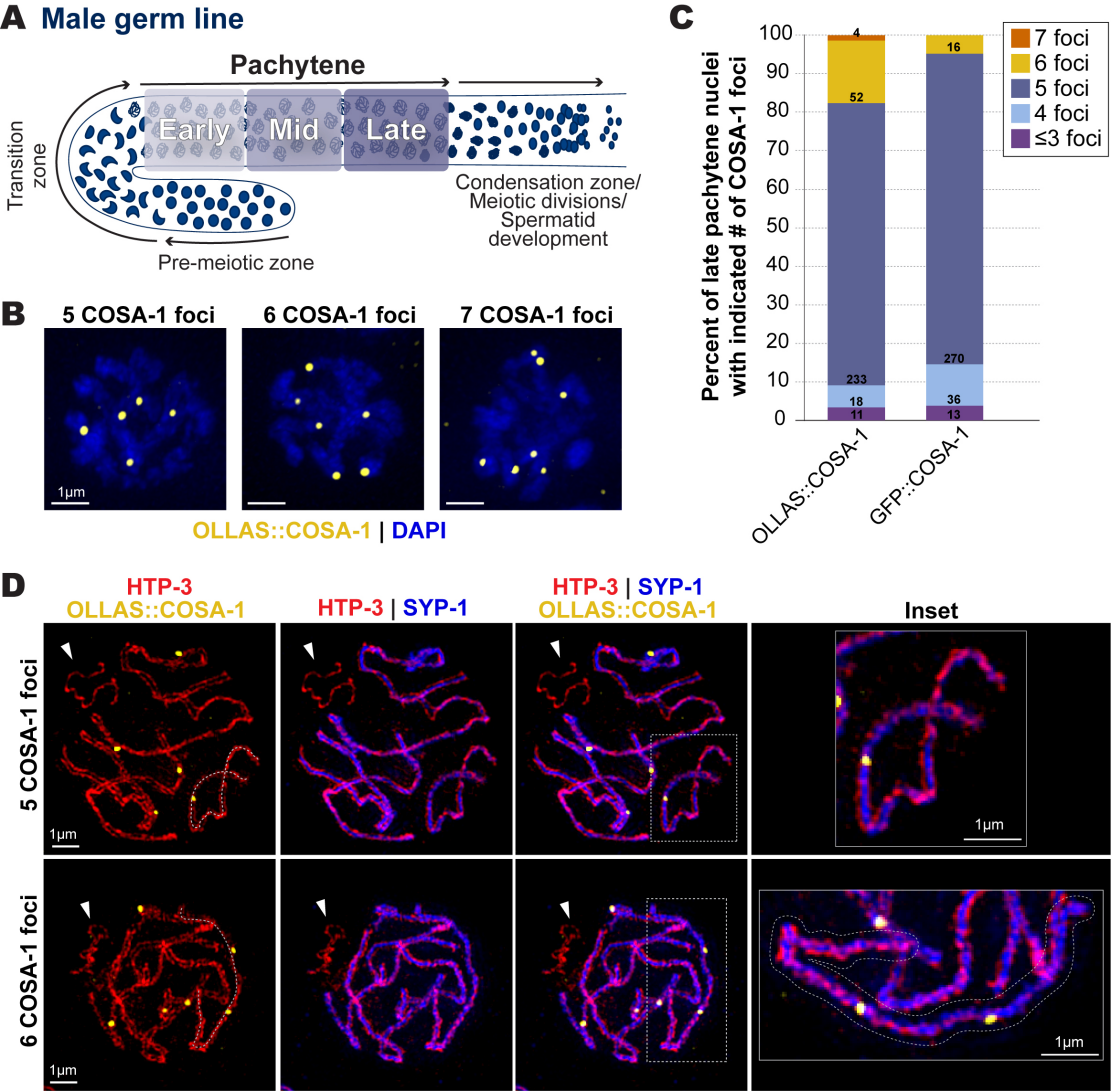
**(A)** Diagram of the *C. elegans* male germ line. Arrows indicate direction of meiotic progression. For this study, we subdivided pachytene into three equally sized regions based on the total length of pachytene: early (first third), mid (middle third), and late (last third) (See Methods). **(B)** Representative images of late pachytene nuclei with 5, 6, or 7 OLLAS::COSA-1 foci (yellow). **(C)** Bar graph indicating the percentages of late pachytene nuclei with ≤3, 4, 5, 6, or 7 COSA-1 foci detected in strains expressing either OLLAS::COSA-1 or GFP::COSA-1. The numbers within each bar indicate the numbers of nuclei in each category from a total of 8 germ lines for OLLAS::COSA-1 and 7 germ lines for GFP::COSA-1. **(D)** Representative SIM images depicting spermatocyte chromosome spreads from late pachytene nuclei with 5 or 6 OLLAS::COSA-1 foci (yellow), HTP-3 (red, chromosome axis), and SYP-1 (blue, synaptonemal complex). White arrowheads indicate the unsynapsed partnerless *X* chromosome. The dashed line in the first column traces the path of a single synapsed chromosome pair with either one COSA-1 focus (top) or two COSA-1 foci (bottom). The chromosome pair with two COSA-1 foci is outlined in the inset shown at the right.

## Description

During egg and sperm development in many organisms, homologous chromosome pairs must establish a crossover to ensure their proper orientation toward and segregation to opposite spindle poles during the first meiotic division. Despite the importance of crossovers for both spermatocyte and oocyte meiosis in most species, the number and distribution of crossovers along chromosomes is often sexually dimorphic (Drouaud *et al.* 2007; Lim *et al.* 2008; Gabdank and Fire 2014; Brick *et al.* 2018; Cahoon and Libuda 2019; Capilla-Perez *et al.* 2021). From genetic assays performed in *C. elegans* oocytes, each of the 6 homologous chromosome pairs typically undergoes only a single crossover, with double crossover meiotic products reported as very infrequent (Hodgkin *et al.* 1979; Nabeshima *et al.* 2004; Lim *et al.* 2008; Hayashi *et al.* 2010; Gabdank and Fire 2014; Li *et al.* 2018). Cytological assays in oocytes using a GFP tagged version of the pro-crossover protein COSA-1 (Yokoo *et al.* 2012) are consistent with the genetically-detected incidence of crossovers; 6 GFP::COSA-1 foci are detected in late pachytene nuclei, which reflects the accumulation of COSA-1 at a single crossover-designated site forming on each of the 6 homologous chromosome pairs (bivalents) (Yokoo *et al.* 2012; Libuda *et al.* 2013). Based on genetic assays detecting meiotic crossovers on Chromosomes *I* , *IV* , and *V,* double crossover events appear to occur more frequently in *C. elegans* male ( *XO* ) spermatocytes than in *C. elegans* hermaphrodite ( *XX* ) oocytes (Hodgkin *et al.* 1979; Henzel *et al.* 2011; Gabdank and Fire 2014; Li *et al.* 2020). Genetic assays have detected double-crossover meiotic products in spermatocyte meiosis at frequencies of 2.7%, 4.4%, and 1.7% for chromosomes *I* , *IV* , and *V,* which would imply double-crossover bivalents occurring at frequencies of 10.8%, 17.6% and 6.8%, respectively (Hodgkin *et al.* 1979; Gabdank and Fire 2014; Li *et al.* 2020). Based on these genetic data, cytological assays using markers such as COSA-1 might be expected to detect nuclei with one or more bivalents harboring two crossover-site foci at frequencies around 40%. However, cytological assessment of GFP::COSA-1 foci in spermatocyte meiosis has detected fewer nuclei with apparent double-crossover bivalents than predicted by the genetic data (Li *et al.* 2020), prompting us to evaluate whether different epitope tags on COSA-1 might influence the number of cytologically-detected double crossover events in spermatocyte meiosis.

We used immunofluorescence to visualize crossover-designated sites in spermatocytes using two different tagged versions of COSA-1: (1) GFP::COSA-1 expressed from a chromosomally-integrated transgene construct using the *pie-1* promotor and the *cosa-1* 3’ UTR (Yokoo *et al.* 2012); and (2) OLLAS::COSA-1 expressed from the endogenous *cosa-1* locus modified using CRISPR/Cas9 to introduce the OLLAS tag (Janisiw *et al.* 2018). In oogenesis, both tagged versions of COSA-1 produce similar results displaying 6 COSA-1 foci in late pachytene (Janisiw *et al.* 2018). In spermatocytes, we found that a majority of nuclei displayed 5 COSA-1 foci with both tagged versions of COSA (Figure 1B,C), which is consistent with previous reports of a single crossover-designated site on each of the 5 autosome pairs (and none on the partnerless *X* chromosome) (Li *et al.* 2020). Further, the fraction of nuclei in which we detected >5 GFP::COSA-1 foci per nucleus (4.8%) was comparable to that observed by Li *et al.,* who used native GFP fluorescence to detect GFP::COSA-1 expressed from a CRISPR-modified endogenous *cosa-1* locus (P = 0.14, Fisher’s Exact test) (Li *et al.* 2020). However, we found that the fraction of late pachytene nuclei with >5 OLLAS::COSA-1 foci (17.6%) was significantly higher than the fraction of late pachytene nuclei with >5 GFP::COSA-1 foci (Figure 1B,C; P<0.0001, Fisher’s Exact test). This difference in the incidence of late pachytene spermatocyte nuclei with >5 tagged COSA-1 foci may reflect: 1) differences in detection sensitivity for the two markers, potentially resulting from the presence of untagged COSA-1 protein expressed from the endogenous *cosa-1* locus in the strain with the *gfp::cosa-1* transgene; and/or, 2) COSA-1 tag-specific effects on crossover designation in spermatocytes. Future studies examining spermatocyte COSA-1 foci, which are not uniform in size or intensity (Figure 1C), may provide insights into how specific tags influence the detection of COSA-1 foci.

We used super-resolution Structured Illumination Microscopy (SIM) combined with meiotic chromosome spreads (Woglar and Villeneuve 2018) to determine whether nuclei with >5 OLLAS::COSA-1 foci display single synapsed bivalents harboring two crossover-site foci. To visualize chromosome structures, we used antibodies detecting the chromosome axis protein HTP-3 (which forms two parallel tracks, one on each homologous chromosomes) and the synaptonemal complex protein SYP-1 (which forms a single track between the two HTP-3 tracks). This analysis confirmed the presence of individual synapsed bivalents with two OLLAS::COSA-1 foci (Figure 1D). Further, we did not observe OLLAS::COSA-1 foci on the unsynapsed hemizygous *X* chromosome, which assembles chromosome axes containing HTP-3 but does not load SYP proteins (Figure 1D) (Jaramillo-Lambert and Engebrecht 2010), consistent with previous work indicating a lack of designated crossover sites on the *X* chromosome in male spermatocytes (Checchi *et al.* 2014). Taken together, our results indicate that: 1) chromosome pairs harboring two crossover-designated recombination events in spermatocyte meiosis can be visualized using OLLAS::COSA-1; and, 2) COSA-1 exhibits epitope tag-specific differences in the detection of double crossover designated sites.

## Methods

All worm strains were generated from N2 backgrounds and maintained as mating stocks at 20 **°** C under standard conditions on nematode growth media (NGM) with lawns of *Escherichia coli* ( *E. coli* ).

*Immunohistochemistry and Chromosome Spreads*

Immunofluorescence was performed as described in (Libuda *et al.* 2013; Cahoon and Libuda 2021). A detailed protocol can be found here: https://www.protocols.io/view/immunofluorescence-for-c-elegans-gonads-xrgfm3w (Roelens 2019). Chromosome spreads were performed in low salt dissection solution (0.1% v/v Tween-20 and 10% v/v Hank’s Balanced Salt Solution (Gibco, 24020117) on an ethanol-washed Superfrost Microscope Slide (Fisher Scientific, 12-544-7) and were then prepared as described in (Pattabiraman *et al.* 2017).

*Microscopy*

Immunofluorescence slides of fixed gonad stained for GFP::COSA-1 or OLLAS::COSA-1 were imaged on a GE DeltaVision microscope with a 63x/N.A. 1.42 lens and 1.5x optivar at 1024x1024 pixel dimensions. Images were acquired using 0.2 µm Z-step size and deconvolved with softWoRx 7.2.2 deconvolution software. 3D-Structured Illumination Microscopy (SIM) was performed as described in (Pattabiraman *et al.* 2017) on a GE DeltaVision OMX Blaze microscopy system (Version 3.70.9622.0) at the Stanford University Cell Sciences Imaging Core Facility (RRID:SCR_017787). Images were acquired with a 100x/N.A. 1.40 oil objective at 512x512 pixel dimensions using 0.125 µm Z-step size. Images were SIM reconstructed and registration corrected with softWoRx. All images were max intensity Z-projected and slightly adjusted for brightness and contrast.

*Quantification of COSA-1 foci*

Imaged gonad were stitched together using the FIJI (NIH) plugin Stitcher (Release 3.1.9; Preibisch *et al.* 2009) and analyzed in Imaris 9.9 as described in (Toraason *et al.* 2021) with minor changes. The start of the pachytene zone was defined by the first row of nuclei that did not contain more than 1-2 nuclei with DNA in a polarized or crescent shape organization characteristic of the transition zone. The end of the pachytene zone was defined as the last row that contained predominantly pachytene nuclei with ≤ 1 nucleus exhibiting condensation zone nuclear organization (Shakes *et al.* 2009). The calculated length of the pachytene region was normalized per germline from 0 to 1. Then, the normalized pachytene length was equally divided into thirds and the nuclei within the last third (length: 0.67-1.0) were defined as late pachytene.

*Statistical Analysis*

Fisher’s Exact statistical analysis was performed using GraphPad QuickCalcs (2023 GraphPad Software). The P value and test used is indicated in the Description section. All n values for nuclei and germlines scored are listed in the figure or figure legend.

## Reagents

**Table:** 

**Strain**	**Genotype**	**Available from**
AV630	*meIs8[unc-119(+) pie-1promoter::gfp::cosa-1] II.*	CGC
NSV97	*cosa-1[ddr12(OLLAS::cosa-1)] III.*	(Janisiw *et al.* 2018)

**Table:** 

**Antibody**	**Animal and Clonality**	**Description**	**Source**
Anti-OLLAS	Rabbit, polyclonal	Use at 1:1,000 - 1:1,500	Genscript, A01658
Anti-GFP booster Alexa Fluor 488	N.A.	Use at 1:200	Chromotek, gb2AF488-50
Anti-SYP-1	Guinea Pig, polyclonal	Use at 1:200	(MacQueen *et al.* 2002)
Anti-HTP-3	Chicken, polyclonal	Use at 1:500	(MacQueen *et al.* 2005)
Anti-Chicken Alexa Fluor 405	Goat, polyclonal	Use at 1:200	Abcam, Ab175675
Anti-Rabbit Alexa Fluor 488 (F(ab)’2 fragment)	Goat, polyclonal	Use at 1:1,000	ThermoFisher, A11070
Anti-Guinea Pig Alexa Fluor 555	Goat, polyclonal	Use at 1:1,000	ThermoFisher, A21435
Anti-Rabbit Alexa Fluor 488 (whole antibody)	Goat, polyclonal	Use at 1:200	ThermoFisher, A11034
